# Glycan Specificity of P[19] Rotavirus and Comparison with Those of Related P Genotypes

**DOI:** 10.1128/JVI.01494-16

**Published:** 2016-10-14

**Authors:** Yang Liu, Theresa A. Ramelot, Pengwei Huang, Yan Liu, Zhen Li, Ten Feizi, Weiming Zhong, Fang-Tzy Wu, Ming Tan, Michael A. Kennedy, Xi Jiang

**Affiliations:** a Division of Infectious Diseases, Cincinnati Children's Hospital Medical Center, Cincinnati, Ohio, USA; b Department of Chemistry and Biochemistry, Miami University, Oxford, Ohio, USA; c Glycosciences Laboratory, Department of Medicine, Imperial College London, London, United Kingdom; d Center for Research, Diagnostics and Vaccine Development, Centers for Disease Control, Taipei, Taiwan; e Department of Pediatrics, University of Cincinnati College of Medicine, Cincinnati, Ohio, USA; Instituto de Biotecnologia/UNAM

## Abstract

The P[19] genotype belongs to the P[II] genogroup of group A rotaviruses (RVs). However, unlike the other P[II] RVs, which mainly infect humans, P[19] RVs commonly infect animals (pigs), making P[19] unique for the study of RV diversity and host ranges. Through *in vitro* binding assays and saturation transfer difference (STD) nuclear magnetic resonance (NMR), we found that P[19] could bind mucin cores 2, 4, and 6, as well as type 1 histo-blood group antigens (HBGAs). The common sequences of these glycans serve as minimal binding units, while additional residues, such as the A, B, H, and Lewis epitopes of the type 1 HBGAs, can further define the binding outcomes and therefore likely the host ranges for P[19] RVs. This complex binding property of P[19] is shared with the other three P[II] RVs (P[4], P[6], and P[8]) in that all of them recognized the type 1 HBGA precursor, although P[4] and P[8], but not P[6], also bind to mucin cores. Moreover, while essential for P[4] and P[8] binding, the addition of the Lewis epitope blocked P[6] and P[19] binding to type 1 HBGAs. Chemical-shift NMR of P[19] VP8* identified a ligand binding interface that has shifted away from the known RV P-genotype binding sites but is conserved among all P[II] RVs and two P[I] RVs (P[10] and P[12]), suggesting an evolutionary connection among these human and animal RVs. Taken together, these data are important for hypotheses on potential mechanisms for RV diversity, host ranges, and cross-species transmission.

**IMPORTANCE** In this study, we found that our P[19] strain and other P[II] RVs recognize mucin cores and the type 1 HBGA precursors as the minimal functional units and that additional saccharides adjacent to these units can alter binding outcomes and thereby possibly host ranges. These data may help to explain why some P[II] RVs, such as P[6] and P[19], commonly infect animals but rarely humans, while others, such as the P[4] and P[8] RVs, mainly infect humans and are predominant over other P genotypes. Elucidation of the molecular bases for strain-specific host ranges and cross-species transmission of these human and animal RVs is important to understand RV epidemiology and disease burden, which may impact development of control and prevention strategies against RV gastroenteritis.

## INTRODUCTION

Rotaviruses (RVs) are the principal cause of severe diarrhea in children, responsible for approximately 200,000 deaths in children younger than 5 years old worldwide in 2011 (1–3). It has been shown that RV attachment to cell surface carbohydrates, mediated by the VP8* domain of the VP4 spike protein, is the important first step for successful infection (4–7). RVs are diverse in recognizing different receptors, causing infections in humans and different animal species. For example, early studies showed that infection with some animal RVs relies on the terminal sialic acid (SA) and that these animal RVs are sialidase sensitive, but the majority of human and animal RVs are sialidase insensitive (8–12). However, a recent study indicated that a sialidase-insensitive human RV, Wa (P[8]), recognizes an internal SA residue (13). In addition, other host cell surface molecules, such as heat shock cognate protein 70 (14–16) and integrins (17–19), have been identified as potential RV receptors, although their precise roles in RV attachment, penetration, and pathogenesis in host cells remain elusive.

The recent findings that almost all major RV genotypes in genogroups P[II] to P[IV] infect humans and recognize histo-blood group antigens (HBGAs) (20–27) have led to the plausible hypothesis that HBGAs are important host factors or cellular receptors for human RVs. Direct evidence for HBGA-RV interaction has been obtained by resolving the VP8* protein crystal structures for two human RVs (P[14] and P[11]) in complex with their HBGA oligosaccharide ligands (21, 22). The association between RV infection and a child's secretor status has also been observed through epidemiologic studies (28–31). Together, these findings suggest that HBGAs play important roles in RV infection and pathogenesis.

Although direct cocrystal data remain lacking, recognition of HBGAs by the most prevalent human rotavirus genotypes, P[4], P[6], and P[8], has been established by *in vitro* binding assays. For example, the VP8*s of the human P[8] and P[4] RVs recognize the Lewis b (Le^b^) and H-type 1 HBGAs, while the P[6] RVs bind the H type 1 only (23). However, a saturation transfer difference nuclear magnetic resonance (STD NMR)-based study showed that while the human P[4] (strain DS1) and P[6] (strain RV-3) RV VP8* proteins could bind the A-type HBGAs with the involvement of the α1-2 fucose, the VP8* of the P[8] human strain Wa did not recognize the A or the Lewis b/H-type 1 antigens (20). On the other hand, a binding study with a human milk glycan library confirmed the binding of the P[6] (RV-3) VP8* to the terminal type 1 HBGA sequences with or without the H epitope but without the Lewis epitope and the involvement of an additional internal Lewis x determinant (32).

**FIG 1:**
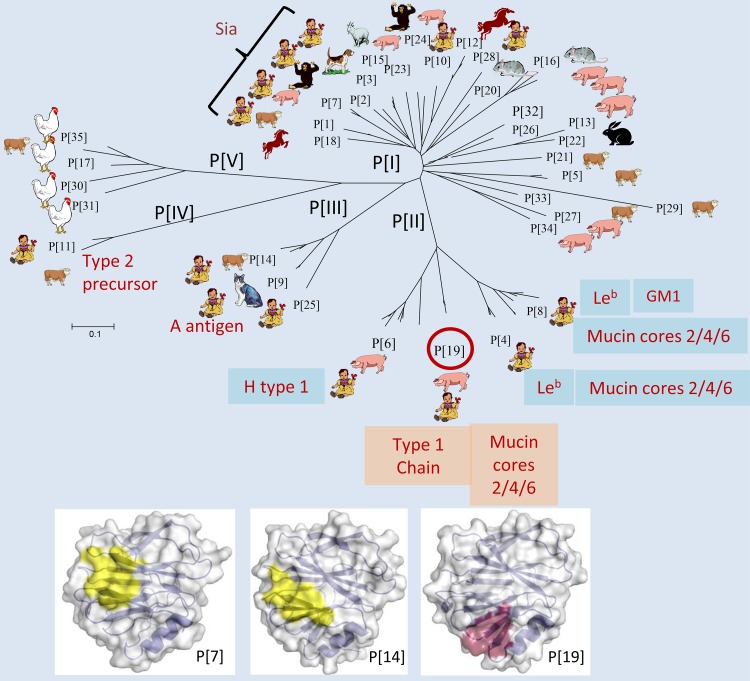
Phylogeny, host ranges, and potential host glycan ligands for group A rotaviruses. The group A RVs have been divided into 37 P genotypes (P[1] to P[37]) based on the major surface spike protein VP4 (33). A phylogeny dividing 35 group A RVs (P[1] to P[35]) into five P genogroups (P[I] to P[IV]), reported previously (25), is shown. The assigned human and animal hosts were based on the ranks of VP4 or VP8* sequence numbers reported for individual species, with only the top (internal row) and second (outer row) ranks shown. It is noted that limited numbers of sequences were reported for genotypes P[10], P[18], and P[20] as well as the newly identified genotypes P[28] to P[35], and the true host species for these genotypes need to be verified in future studies. The carbohydrate ligands recognized by genotypes reported in the literature are listed, including P[1], P[2], P[3], and P[7] (9); P[4], P[6], and P[8] (13, 20, 23); P[9], P[14], and P[25] (25); P[11] (24, 26); and P[19] (this study). Comparison of the P[7] (PDB ID 2I2S [44]) and P[14] (PDB ID 4DRV [21]) receptor binding sites with that of P[19] is in the bottom portion. The figure is modified based on data presented in previously published references (21–23, 25, 44).

The group A RVs are diverse and have been divided into 37 P genotypes (P[1] to P[37]) in five P genogroups (P[I] to P[V]) based on sequences of the viral surface spike proteins VP4 and VP8* (25, 33) (Fig. 1). While the majority of RVs exclusively or nearly exclusively infect either humans or particular animal species, others appear to commonly infect both humans and animals (P[6], P[9], P[11], and P[14] in P[II] to P[IV]) or infect mainly animals, with occasional infections of both humans and animals (P[1], P[2], P[3], P[7], P[10], and P[28] in P[I]) (Fig. 1). Mechanisms for cross-species transmission have been suggested for some RVs, such as the P[III] RVs that can infect both humans and animals, probably via recognition of the A antigens shared by humans and wild and domestic animals (25). In addition, the P[11] RVs recognize HBGA precursors or backbones whose chain length and branching are developmentally controlled in neonates and young infants (24, 26). These carbohydrates are evolutionarily conserved between humans and some animals. However, the mechanisms of P[1], P[2], P[3], P[7], P[10], and P[28] RV infection in animals or occasionally both human and animals remain unknown, limiting our understanding of RV epidemiology and, thus, the ability to develop rational prevention strategies.

In this study, we characterized a P[19] RV that commonly infects animals (pigs) but rarely humans (Fig. 1). A unique binding property to the disaccharide core structure (GlcNAcβ1-6GalNAc) of mucin cores 2, 4, and 6 and the extended terminal type 1 HBGA trisaccharide (Galβ1-3GlcNAcβ1-3Gal) was identified for an overlapping binding interface. This P[19] RV binding interface differed from known ones but appeared to be conserved among P[II] RVs and two P[I] RVs (P[10] and P[12]) (Fig. 1). Additional saccharides surrounding the core glycan sequences also impact the binding specificities for these RVs. This may explain the observed diversity in binding specificity, host range, and possibly pathogenicity of RVs in different human populations and animal species.

## MATERIALS AND METHODS

### Expression and purification of VP8* proteins in Escherichia coli.

The VP8* sequences (amino acids 46 to 231) from a human RV P[19] (GenBank accession number DQ887060) was expressed in E. coli BL21 and purified by glutathione *S*-transferase (GST) tag affinity purification, as described previously (25). Recombinant VP8* proteins of other P[II] RVs (P[4], P[6], and P[8]) and a P[10] RV were also purified using the same expression system. ^15^N- or ^15^N,^13^C-labeled P[19] VP8* proteins were also made for the NMR studies using the same E. coli culture procedures, with the addition of ^15^N-minimal growth medium (22 mM KH_2_PO_4_, 50 mM Na_2_HPO_4_, 8.5 mM NaCl, 0.1 mM CaCl_2_, 2 mM MgSO_4_, 1× BME vitamins (catalog no. B6891; Sigma), 18 mM ^15^NH_4_Cl, and 22 mM glucose) or ^15^N,^13^C-minimal growth medium supplied with 22 mM [^13^C]glucose (20). The GST tag of the labeled VP8* proteins was removed for the NMR study by cutting with thrombin (Sigma-Aldrich Co., St. Louis, MO), followed by fast protein liquid chromatography (FPLC) gel filtration to collect the VP8* protein fractions and concentration by ultracentrifugation. The purified VP8* proteins were stored at −80°C until needed.

### Binding of P[19] VP8* to human saliva, human milk, and porcine mucins.

A set of previously characterized adult saliva samples with known ABO, secretor, and Lewis phenotypes were used in binding assays with the GST-VP8* fusion proteins (23). In addition, a set of saliva samples from neonates and infants at different ages in the first year of life described previously (24) was studied for age-specific host ranges of P[6] RVs. A pooled human milk sample from the Cincinnati human milk bank (34) and a commercial porcine mucin (Sigma) were also tested for binding to P[19] VP8*. The carbohydrate profiles for the human milk and porcine mucin have been determined in our previous studies (25, 34).

### Glycan array screening and enzyme-linked immunosorbent assay (ELISA)-based oligosaccharide binding assay.

Initial ligand screenings for P[19] VP8* was performed by the Protein-Glycan Interaction Core of the Consortium for Functional Glycomics (CFG) (the glycan library information was available from the website http://www.functionalglycomics.org/). The recombinant GST-VP8* proteins were applied to individual glycan arrays at a protein concentration of 200 μg/ml, and the bound GST-VP8* proteins were detected using a fluorescence-labeled anti-GST monoclonal antibody. Relative fluorescent units (RFU) of each glycan were calculated to rank the reactivity in the interaction with P[19] VP8*. A further microarray analysis was performed at the carbohydrate microarray facility of the Glycosciences Laboratory at the Imperial College, London, United Kingdom, using a focused array of sequence-defined glycan probes arrayed as lipid-linked probes (35). The probes were prepared from reducing oligosaccharides by reductive amination with the amino lipid 1,2-dihexadecyl-*sn*-glycero-3-phosphoethanolamine, designated DH. The P[19] VP8* was overlaid at 50 μg/ml, followed by rabbit anti-GST antibody Z-5 (Santa Cruz, Dallas, TX) and biotinylated anti-rabbit IgG (Sigma). Binding was detected with Alexa Fluor 647-labeled streptavidin (Molecular Probes, Eugene, OR). The probes have been described previously (36, 37) except for DFiLNO, a difucosylated decasaccharide received from Dextra as an unknown impurity of TFiLNO (1-2,-2,-3) and characterized by negative-ion electrospray tandem mass spectrometry with collision-induced dissociation (ES-CID-MS/MS) (W. Chai, unpublished data).

The ELISA-based oligosaccharide binding assays were carried out as previously described (38). Oligosaccharides used in the present study included biotin-labeled polyacrylamide (PAA) conjugates obtained from CFG and Glycotech (GlycoTech, Inc., Gaithersburg, MD) and biotin-labeled BSA conjugates of A, B, and H type 1 to type 4, kindly provided by Todd L. Lowary.

### 2D ^1^H-^15^N-HSQC NMR experiments.

Titrations of P[19] VP8* with mucin core 2 (kindly provided by James Paulson) and LNFPI (V-lab) were followed by two-dimensional heteronuclear single quantum correlation (2D ^1^H-^15^N-HSQC) experiments and used to calculate solution dissociation constants (*K_d_*s) for binding of the two ligands to P[19] VP8*. The *K_d_*s were defined as [VP8*][ligand]/[VP8*-ligand], where [VP8*-ligand] refers to the concentration of the complex of VP8* and the glycan ligand and [VP8*] and [ligand] are the concentrations of free protein and ligand. NMR data were collected at 20°C with samples of ∼300 μl of Tris NMR buffer (10 mM Tris-HCl, 100 mM NaCl, 1 mM EDTA, 10% D_2_O [pH 8.0]) in 5-mm Shigemi NMR tubes on a 600-MHz Bruker Avance III spectrometer. Samples of 0.25 mM ^15^N-labeled VP8* were titrated with each ligand at 0, 0.1, 0.3, 0.5, 1.0, 1.4, 1.9, 2.7, 4.3, and 6 mM, and 2D ^1^H-^15^N-HSQC spectra were acquired after each titration step. Spectra were processed with NMRPipe software (39) and analyzed with Sparky (http://www.cgl.ucsf.edu/home/sparky). The weighted chemical shift change (CSC) upon titration was determined for 10 strongly affected backbone amide peaks that were not overlapped according to the following formula (40): CSC = Σ {[(ΔδH^N^)^2^ + (ΔδN/6.5)^2^]}^1/2^, ΔδH^N^ = δH^N^_free_ − δH^N^_bound_, and ΔδN = δN_free_ − δN_bound_, where δN_free_ and δN_bound_ are the amide ^15^N chemical shifts in the absence and presence of ligand and δH^N^_free_ and δH^N^_bound_ are the backbone amide hydrogen chemical shifts, respectively. The concentration of free ligand, [L-free], was determined by the equation [L-free] = [L-total] − (1/*f*) × [P-total], where *f* is the fraction of VP8* in the bound state is CSC/CSC-max, and [L-total] and [P-total] are the total concentrations of ligand and protein at each titration. The CSC-max and *K_d_*s were determined by fitting the CSC/CSC-max at each ligand titration versus the [L-free] to the equation *f* = [L-free]/(*K_d_* + [L-free]), with the variables CSC-max and *K_d_*. The [L-free] values were calculated with the updated CSC-max, and this process was repeated until there were no further changes. The error bars for *K_d_* were determined based on the average and standard deviation of the 10 peaks that had their CSC/CSC-max versus [L-free] plotted and fit to *K_d_* in the same way.

### P[19] VP8* chemical shift assignments.

In order to assign the backbone chemical shifts, 2D and 3D NMR data were collected for ∼300 μl of 1.1 mM ^15^N-^13^C-labeled P[19] VP8* in Tris NMR buffer at 20°C on 600-MHz Varian Inova, 600-MHz Bruker Avance III, and 850-MHz Bruker Avance II NMR spectrometers equipped with conventional 5-mm HCN probes. Backbone ^1^H, ^13^C, and ^15^N resonance assignments were made using the PINE server and then corrected and completed manually based on the following 3D spectra (41): HNCACB, CBCA(CO)NH, HNCO, HNCA, HN(CO)CA, HN(CA)CO, HBHA(CO)NH, and ^15^N-edited NOESY-HSQC (τ_m_ = 100 ms). Backbone assignments were 91% complete for residues 63 to 225, and only 5 to 10 HN cross-peaks could not be assigned. However, none of the unassigned peaks shifted in the titration studies.

### Ligand chemical shift assignments.

Chemical shift assignments of mucin core 2 and LNFPI were achieved by analysis of 2D ^1^H-^13^C HSQC, heteronuclear multiple-bond correlation (HMBC), HSQC-total correlation spectroscopy (HSQC-TOCSY), ^1^H-^1^H correlation spectroscopy (COSY), TOCSY, and nuclear Overhauser effect spectroscopy (NOESY), and 1D ^1^H NMR experiments recorded at 20°C and referenced to internal trimethylsilylpropanoic acid (TSP) (20, 42). The NMR samples were 2 mM mucin core 2 in Tris NMR buffer and 5 mM LNFPI in phosphate NMR buffer (20 mM sodium phosphate, 100 mM NaCl, 10% D_2_O), both at pH 8.0. Chemical shift assignments for LNFPI were in agreement with published values (43), although differences can be attributed to pH, temperature, and referencing variations.

### STD NMR experiments.

All saturation transfer difference (STD) NMR spectra were acquired in Shigemi Tubes (Shigemi, USA) with a Bruker 600-MHz Avance III spectrometer at 10°C using a conventional HCN probe and the TopSpin 3.1 pulse sequence STDDIFFGG19.3. The NMR sample for mucin core 2 in complex with P[19] VP8* was prepared with 200 μM protein and 6.0 mM ligand in a final volume of 300 μl of Tris NMR buffer. The LNFPI STD sample was prepared with 130 μM P[19] VP8* and 6.6 mM LNFPI in a final volume of 300 μl of phosphate NMR buffer. This results in total protein-ligand ratios of 1:35 and 1:50 for the mucin core 2 and LNFPI samples, respectively. The protein resonances were saturated at 0 ppm with a cascade of 40 Gaussian-shaped pulses with duration of 50 ms, resulting in a total saturation time of 2 s. The off-resonance saturation was applied to 30 ppm, and 1,024 experiments were acquired for each with a 5-s recycle delay. A spin-lock filter with strength of 10 kHz and duration of 100 to 200 ms was applied to suppress the broad protein resonance signals, and Watergate 3-9-19 was used to suppress the residual water signal. The STD spectra were obtained by subtracting the on-resonance spectrum from the off-resonance reference spectrum. STD amplification factors were determined by the equation (*I*_o_ − *I*_sat_)/*I*_o_, where *I*_o_ and *I*_sat_ are the intensities of the signals in the reference and saturated spectra, respectively, and the normalized STD amplification percentage was determined by dividing by the largest STD amplification factor for each ligand. The protons with the largest STD amplification percentage along with data from the 2D STD TOCSY spectrum, which were used to resolve overlap from the 1D spectra, were used to assign the binding epitopes.

### Homology modeling of P[19] VP8* and site-directed mutagenesis.

A homology model of the P[19] VP8* protein was built based on the X-ray crystal structure of the DS1 strain of RV (PDB identifier [ID] 2AEN) as the template by SWISS-MODEL automated protein structure homology-modeling server (http://swissmodel.expasy.org/). Mutant VP8* proteins with single amino acid substitutions were constructed from template wild-type VP8* constructs using the QuikChange site-directed mutagenesis kit (Stratagene, La Jolla, CA).

### Virus propagation, inhibition, and infectivity assays.

A P[19] RV (G9P[19]) was adapted from a positive stool sample obtained from Taiwan's Centers for Disease Control (CDC) collected on 17 December 2014 by multiple blind passages on MA104 cells. The identity of the strain was confirmed by reverse transcription-PCR (RT-PCR) and sequencing following detection of viral replication in the cell culture by ELISA using a Rotaclone kit (Meridian Bioscience, OH). The cell culture-adapted P[19] RVs were then used for inhibition assays with human milk, neonate saliva, and porcine mucin FPLC fractions as well as the oligosaccharides of LNFPI and mucin core 2 using procedures described previously (24). In brief, the P[19] viruses (at 300 fluorescent focus-forming units [FFU]/10 μl) were preincubated with different inhibition reagents for 30 min. After rinsing twice with serum-free Dulbecco modified Eagle medium (DMEM) and chilling of all reagents and the 24-well plates on ice, duplicated wells of confluent MA104 monolayers were inoculated with the virus-oligosaccharide, virus-saliva, or virus-milk mixtures on ice with continuous rocker platform agitation for 1 h. The inocula were then removed and the cells were washed twice with ice-cold serum-free DMEM. The plates were then placed back in the 37°C incubator for 18 to 20 h prior to quantification of infected cells by immunofluorescence with a rabbit anti-rotavirus antibody followed by a fluorescein isothiocyanate (FITC)-labeled goat anti-rabbit secondary antibody.

## RESULTS

### P[19] VP8* binds human saliva, human milk, and porcine mucin.

**FIG 2:**
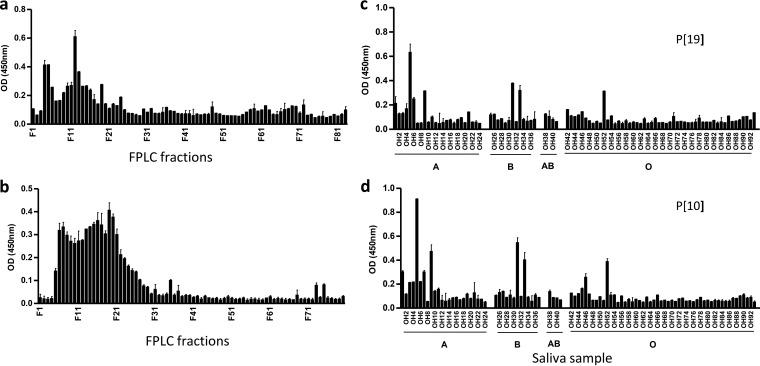
Binding profiles of P[19] VP8* with human milk and porcine mucin and comparison of saliva binding profiles between P[10] and P[19]VP8* proteins. The human milk and porcine mucin samples were fractioned through a Superdex 200 column before testing for binding to P[19] VP8* by following a procedure described previously (24, 25). Binding signals peaked in the high-molecular-weight fractions for both human milk (a) and porcine mucin (b) samples. The P[19] VP8* also revealed a binding profile similar to that of another RV (P[10] in P[I] [d]) among 96 adult human saliva samples tested (c). The ABH and Lewis epitope statuses of the saliva donors are indicated. Each column represents the mean *A*_450_, and the error bars represent the standard deviation for each sample tested in triplicate.

Our study started with *in vitro* binding assays of P[19] VP8* to human milk, human saliva, and porcine mucin for the detection of potential host receptor ligands. The P[19] VP8* proteins bound the high-molecular-weight gel filtration fractions of human milk and porcine mucins (Fig. 2a and b), as well as a group of human saliva samples with variable binding profiles among saliva donors (Fig. 2c), indicating the existence of receptors or ligands in these natural host glycan reservoirs. The pattern of binding of P[19] VP8* to human saliva differed from those of the P[4], P[6], and P[8] RVs (23), where the P[19] VP8* bound a low proportion of samples (12%) with no correlation to the ABH or Lewis types of the saliva donors (Fig. 2c), suggesting that a unique, low-abundance carbohydrate may be responsible for the low prevalence of P[19] in human populations.

### P[19] VP8* recognizes mucin cores and the type 1 HBGA precursor.

**FIG 3:**
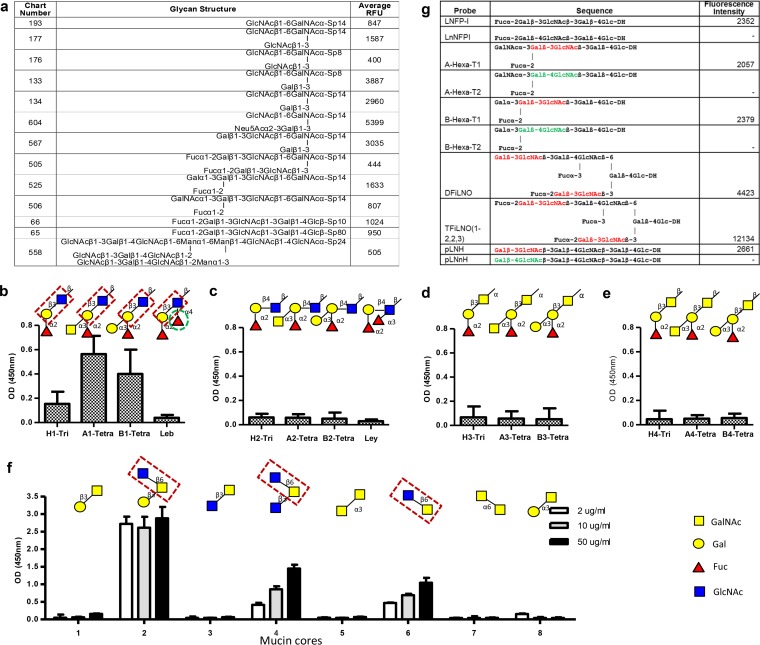
P[19] VP8* recognizes mucin cores 2, 4, and 6 and type 1 HBGAs. (a) A list of top glycans recognized by P[19] VP8* in a glycan array containing 610 glycans. (b to e) Binding to four types (1 to 4) of HBGA glycans, of which P[19] VP8* recognized only type 1 HBGA glycans. The signals for binding to the H1 trisaccharide (Fucα1-2Galβ1-3GlcNAc-) were weak to moderate but significantly increased with the addition of the A or B residues, whereas further addition of a β1-3 fucose (the Lewis epitope, green dashed circle) to the GlcNAc (forming Le^b^) completely blocked the binding. The disaccharide Galβ1-3GlcNAc of the type 1 HBGA precursor (highlighted in red dashed boxes) is believed to be essential for binding, while the terminal fucose, A, or B residues added to Gal [Fucα1-2Galβ1-3GlcNAc-, GalNAcα1-3(Fucα1-2)Galβ1-3GlcNAc-, and Galα1-3(Fucα1-2)Galβ1-3GlcNAc-] could increase the binding. (f) Among the eight mucin core glycans (core 1 to core 8) tested, only mucin cores 2, 4, and 6 revealed binding signals. The disaccharide GlcNAcβ1-6GalNAc is believed to be essential for binding, which is highlighted in red dashed boxes. The ELISA was performed in triplicate, and the whole experiment was repeated once. The error bars represent the standard deviations from triplicate wells. (g) Analysis with a focused array of lipid-linked sequence-defined probes, which was carried out to further determine the HBGA binding specificity of P[19] VP8*. Six pairwise comparisons of type 1 and type 2 chains with different lengths and capping moieties are shown. P[19] VP8* recognized only the type 1-containing probes (red). The type 2 chain probes are marked green. DH represents the amino lipid that the oligosaccharide probes were linked to. A dash indicates fluorescence intensity less than 1.

To define specific carbohydrates recognized by P[19] VP8*, a glycan array analysis was performed by the Consortium of Functional Glycomics (CFG) against a library containing 610 mammalian glycans. Binding signals arose for a group of glycans containing the type 1 HBGAs and/or mucin cores 2, 4, and 6 (Fig. 3a). Further binding assays with different HBGA- and mucin core-related glycans (Fig. 3b to f) confirmed the glycan array results. For example, among the four types (types 1 to 4) of HBGAs tested, the P[19] VP8* bound only the type 1 antigens (Fig. 3b to e). This result was further confirmed by a focused neoglycolipid-based array with pairwise comparisons of oligosaccharide sequences that differed only in linkages between residues in each of the type 1 (Galβ1-3GlcNAc) and type 2 (Galβ1-4GlcNAc) glycans (Fig. 3g). These results indicate that only glycans containing the terminal Galβ1-3GlcNAc sequence were recognized by P[19], further supporting the specificity toward type 1 sequences for P[19].

Additional modifications of the type 1 disaccharide Galβ1-3GlcNAc affected the P[19] VP8* binding signals. For example, the terminal A or B residues, as well as the α1,2-linked fucose, could enhance the binding signals, while the Lewis epitope (α1,4-linked fucose) abolished binding (Fig. 3b). *In vitro* assays of binding of P[19] VP8* to mucin core glycans (mucin cores 1 to 8) also confirmed the glycan array results, with the positive binding signals observed only in those containing the disaccharide GlcNAcβ1-6GalNAc, while further additions of residues GlcNAc β1-3 (mucin core 4) or Gal β1-3 (mucin core 2) linking to GalNAc enhanced the binding (Fig. 3f).

**FIG 4:**
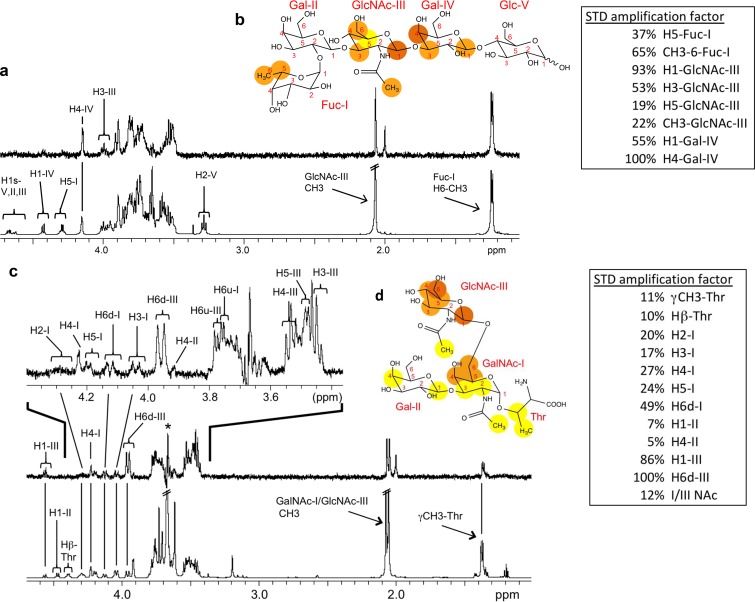
NMR spectra of LNFPI and mucin core 2 in complex with P[19] VP8*. ^1^H NMR of P[19] VP8* (130 μM) with LNFPI (6.6 mM) is shown in panel a. The LNFPI epitope map when bound to P[19] VP8* is shown in panel b. Dark orange, strong STD NMR effects; orange, weaker effects; yellow, weakest STD NMR effects. The STD amplification factors are shown in the box for residues that do not overlap in the 1D STD spectrum. For ^1^H resonances that overlap in the 1D STD spectrum, further 2D STD TOCSY was applied (data not shown). The inclusion of GlcNAc-III H6s and Gal-IV H3 in the binding epitope was based on the 2D STD-TOCSY and colored orange. ^1^H NMR of mucin core 2 (6.0 mM) with P[19] VP8* (200 μM) is shown in panel c, with an expanded spectral view shown at the top. “H6d” indicates a downfield proton, and “H6u” indicates an upfield proton. The epitope map of mucin core 2 when bound to P[19] VP8* is shown in panel d. STD-TOCSY was used to add the overlapping resonances of GlcNAc-III H3/H4/H5 in the binding epitope (data not shown).

The ligand binding specificity of the P[19] VP8* was further characterized by STD NMR. Data for a type 1 HBGA pentasaccharide, LNFPI (Fucα1-2Galβ1-3GlcNAcβ1-3Galβ1-4Glc), demonstrated that the extended type 1 chain of an internal Gal (Fucα1-2Galβ1-3GlcNAcβ1-3Gal) is also involved in the interaction with P[19] VP8*, in addition to the GlcNAc and the terminal (H) fucose (Fig. 4a and b). Similarly, STD NMR of P[19] VP8* with mucin core 2 showed a strong interaction with the disaccharide GlcNAcβ1-6GalNAc, in which the GlcNAc residue was highly involved (Fig. 4c and d).

### P[19] RV replication was inhibited by human saliva, human milk, porcine mucin, and LNFPI glycans.

**FIG 5:**
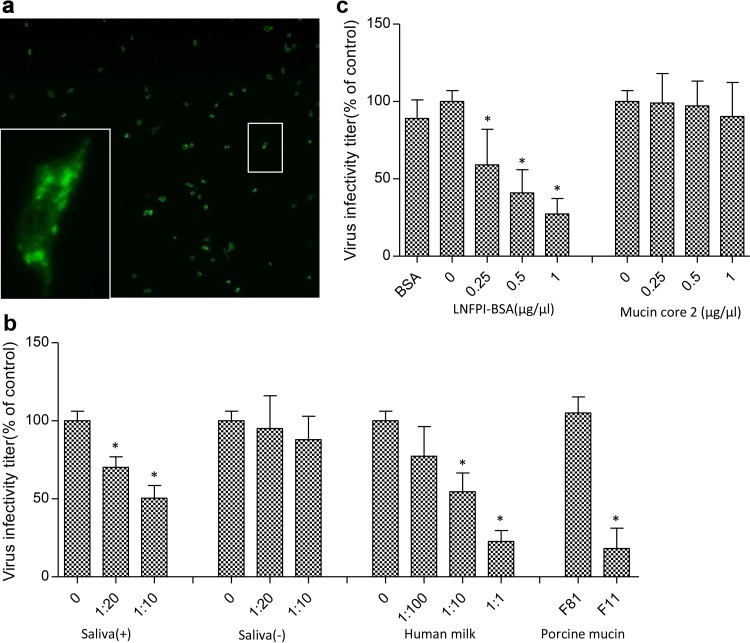
Inhibition of P[19] RV replication in cell culture by human milk, neonatal saliva, porcine mucin, and LNFPI conjugates. (a) Representative immunofluorescence microscopy image of MA104 cells infected with P[19] RV. Each green dot represents an infected cell, as shown in the enlarged panel. The plotted blocking results are shown in panels b and c. The error bars represent standard errors from triplicate repeats, and the experiment was repeated once. The statistical significance was calculated by analysis of variance (ANOVA); the asterisk indicates a statistical difference of <0.05. (b) Blocking results with neonatal saliva, human milk, and porcine mucin FPLC fractions. Human saliva samples that did [saliva (+)] and did not [saliva (−)] bind to P[19] VP8* in the binding ELISAs were used in the blocking assay as positive and negative controls, respectively. The saliva samples were tested at 1:10 and 1:20 dilutions in PBS with a negative control containing PBS only. Serial dilutions of the human milk samples at 1:1, 1:10, and 1:100 were tested. The FPLC fraction of the porcine mucin samples that showed VP8* binding (F11) was tested with an FPLC fraction (F81) that did not bind P[19] VP8* as a negative control. (c) Blocking results with BSA-conjugated LNFPI and free mucin core 2 oligosaccharide at final concentrations of 0.25, 0.5, and 1 μg/μl.

To examine the biologic significance of reactive glycan ligands for P[19] RVs, viral replication inhibition assays were performed on a human P[19] RV. P[19] RV titers were significantly reduced following incubation of the viruses with saliva, porcine mucin fractions (gel filtration), and human milk samples that previously bound P[19] VP8* (Fig. 5a and b). In contrast, the virus titers were not reduced after treatment with samples that did not bind to P[19] VP8* previously (Fig. 5b). Following treatment with human milk, a typical dose-dependent reduction, with an infectivity decrease of up to 77.2%, was observed (Fig. 5b). Similar blocking effects have also been observed for bovine serum albumin (BSA)-conjugated LNFPI (Fig. 5c), whereas the nonconjugated mucin core 2 did not result in an obvious inhibitory effect (Fig. 5c), possibly due to the monovalence of the core 2 oligosaccharide used in the experiment.

### The P[19] VP8* ligand binding interface differed from previously known ones.

**FIG 6:**
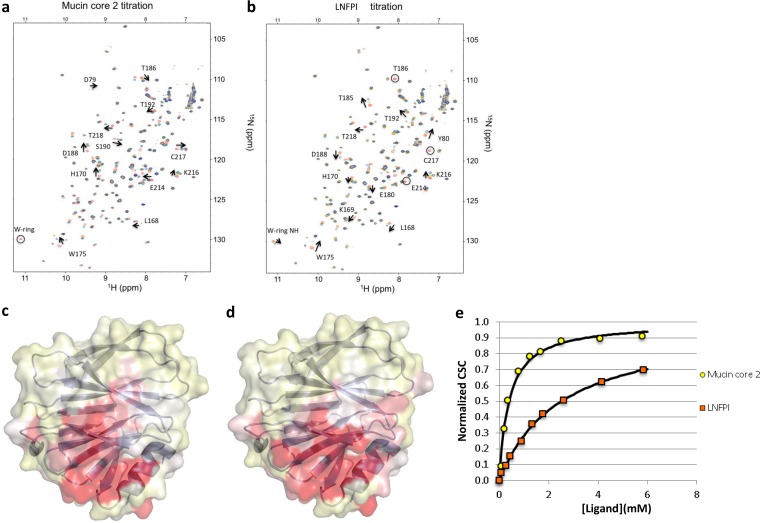
Chemical shift changes in P[19] VP8* upon addition of mucin core 2 (a) or LNFPI (b). Titrations were followed by acquisition of two-dimensional ^1^H-^15^N HSQC spectra of 0.25 mM ^15^N-labeled P[19] VP8* in Tris buffer collected at 20°C on a Bruker Avance III 600-MHz NMR spectrometer. The NMR data correspond to increasing ligand/protein ratios of 0:1 (red), 2:1 (orange), 8:1 (green), and 30:1 (blue). Peaks that disappeared upon titration are circled. Surface representation of the HN chemical shift changes for P[19] VP8* with mucin core 2 (c) and LNFPI (d) is also shown. The data were plotted onto the surface of a P[19] VP8* homology model, with the largest changes in dark red. The chemical shift differences between ^1^H and ^15^N resonances in free and ligand-bound proteins were determined as the weighted chemical shift change. For LNFPI, three HN cross-peaks disappeared upon ligand titration and did not reappear at higher ligand concentrations. These residues, T186, E214, and C217, were set at a maximum Δδ_av_ of 0.25. The 2D ^1^H-^15^N HSQC with a ligand/protein ratio of 30:1 was used for the bound shifts. The protein structure of P[19] VP8* was based on a homology model using the crystal structure of DS1 (a P[4] RV; PDB ID 2AEN) as the template and marked by using the Pymol program. (e) Plot of normalized chemical shift changes (CSC/CSC-max) versus free ligand concentrations.

To explore the glycan binding interface for P[19] RVs, NMR titration studies of mucin core 2 and LNFPI were conducted to measure chemical shift changes in backbone amide resonances followed by ^1^H-^15^N heteronuclear single quantum coherence (HSQC) spectra. All backbone chemical shift files were available from the BioMagResBank database (accession number 25441). Although LNFPI caused slightly more amino acid perturbations, the residues with HSQC chemical shift perturbations for mucin core 2 and LNFPI largely overlapped (Fig. 6a to d), suggesting a common interface for the two ligands. Interestingly, the *K_d_*s of the HSQC chemical shift perturbation for P[19] VP8* binding to mucin core 2 (*K_d_* = 0.4 mM) were smaller than those for binding to LNFPI (*K_d_* = 2.6 mM) (Fig. 6e), indicating that the affinity of P[19] VP8* for mucin core 2 was higher than that for LNFPI. More than half of the residues did not reveal significant perturbations upon addition of each ligand, suggesting that there were no effects due to buffer changes. In addition, NMR of P[19] VP8* with the glycan of LacNAc (Galβ1-4GlcNAc) was conducted as a control experiment, and no clear chemical shifts on P[19] VP8* was observed, which further confirmed the specificity of mucin core 2 and LNFPI for P[19] VP8*.

**FIG 7:**
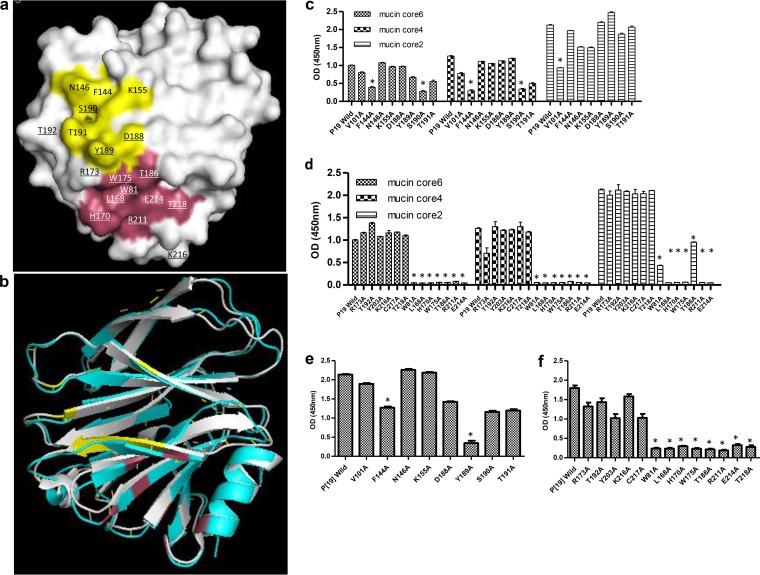
Ligand binding interface for P[19]VP8* confirmed by site-directed mutagenesis. (a) The homology model for P[19] VP8* was constructed based on the DS1 crystal structure (P[4]; PDB ID 2AEN). The surface amino acid residues with chemical shift changes in HSQC NMR titration are underlined. The amino acids corresponding to previously reported S or A antigen binding sites are colored yellow, while the newly identified ligand binding site is colored pink. (b) Superimposition of P[19] VP8* (cyan) with P[3] VP8* (gray; PDB ID 1KQR). The glycan binding interfaces for the two genotypes are colored pink and yellow, respectively. (c) The results for binding of P[19] VP8* and its mutants, each with a signal amino acid mutation at the corresponding SA-binding amino acids (V101, F144, N146, K155, D188, Y189, S190, and T191), to the mucin cores are shown. Mutants with significant glycan binding *A*_450_ changes are marked by an asterisk. (d) The results for binding of P[19] VP8* and its mutants, with chemical shift changes at around the sialic acid or A antigen binding site, to mucin core glycans. Single amino acid mutations of seven residues (W81, L168, H170, W175, T186, R211, and E214) resulted in a complete loss of binding signals. (e) Binding results of the P[19] VP8* and its mutants at the corresponding SA-binding amino acids to the glycan of LNFPI. (f) Binding of mutations corresponding to amino acids that showed chemical shift changes to LNFPI. All seven residues that were found to be involved in mucin core binding (W81, L168, H170, W175, T186, R211, and E214) and an additional residue, T218, also resulted in a complete loss of binding to LNFPI. The binding assay was tested in triplicate, and the entire experiment was repeated twice. The statistical significance was calculated by ANOVA; the mean *A*_450_s with standard deviations are shown.

More than 90% of chemical shift perturbations occurred for a group of amino acids scattered in a location near the previously identified SA- and A-HBGA binding interfaces (11, 21, 44) (Fig. 7a and b). Mutants with single amino acid changes at seven of these perturbed residues (W81, L168, H170, W175, T186, R211, and E214) which could constitute a binding interface of P[19] resulted in complete loss of binding to mucin core glycans (Fig. 7a to d). In addition, besides the above-mentioned seven amino acids, we noted that single amino acid mutations at another two perturbed residues (T218 and Y189) also eliminated binding of P[19] VP8* to LNFPI (Fig. 7e and f). However, mutations at three other perturbed amino acids (D188, Y189, and S190) in the corresponding SA- or A-HBGA binding interface revealed only minor reductions in P[19] binding to mucin cores and LNFPI, while none of other amino acids that constitute the SA- or A-HBGA binding interface had an effect on the binding of P[19] RV to either mucin cores or LNFPI. Together, these data indicate that P[19] uses a new, but related, glycan binding interface that significantly shifted away from the previously known sites. Superimposition comparison of this glycan binding site with those for P[3], P[14], and P[11] supported our observations (Fig. 7b and data not shown).

**FIG 8:**
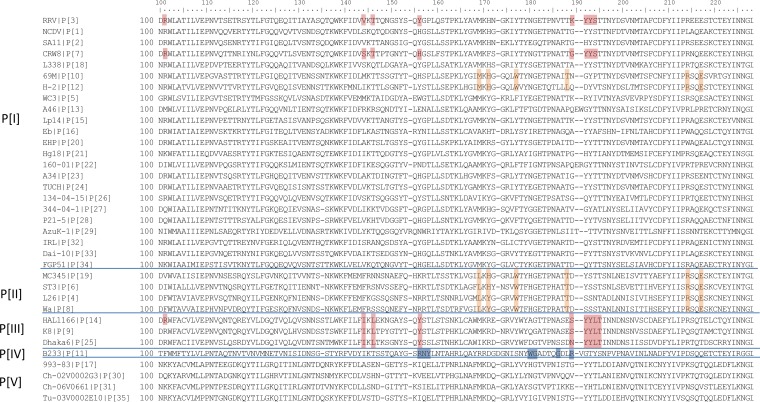
Sequence alignment of VP8*s among 35 [P] genotypes (P[1] to P[35]). One representative VP8* sequence from each of the 35 genotypes is included in the alignment. The amino acids which were deduced to interact with mucin cores and LNFPI glycans in the current study are marked by orange boxes. Residues that interact with sialic acid or type A HBGAs in the corresponding genotypes are marked in red, and those that interact with type 2 HBGAs precursor for P[11] genotype are marked in blue.

Sequence alignment showed that all P[19] strains had identical amino acid compositions on the deduced ligand binding site (data not shown). In addition, alignment of the group A RV VP8* sequences showed that the amino acids of the deduced ligand binding interface of the P[19] RVs are conserved among all other three P[II] RVs (P[4], P[6], and P[8]) (Fig. 8), suggesting that a common host factor or receptor may be responsible for maintenance of the P[II] genogroup. In addition, among the diverse P[I] genogroup RVs that cause diseases in different animal species, we noticed that two genotypes (P[10] and P[12]) also showed a good amino acid conservation of the deduced ligand binding interface. In particular, a single residue, W175, was found to be conserved among two P[I] (P[10]/P[12]) and all P[II] RVs, suggesting an evolutionary connection among these human and animal RVs.

### Similar but genotype-specific glycan binding profiles among P[II] RVs.

**FIG 9:**
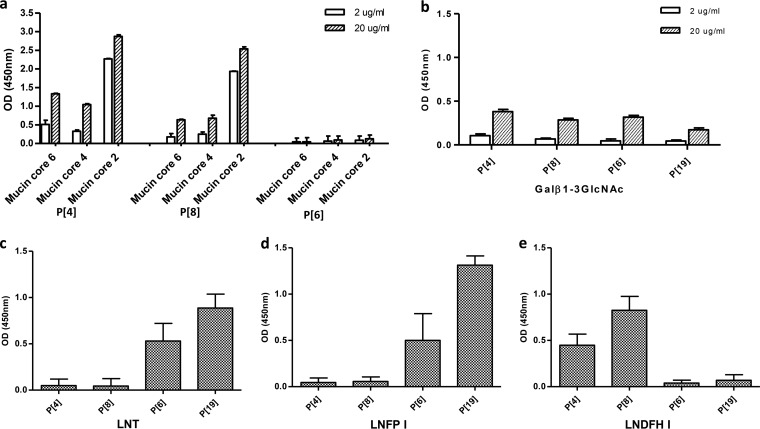
Binding of P[II] RV VP8*s to mucin cores 2, 4, and 6 and type 1 HBGA-related glycans. (a) Binding of the P[4], P[6], and P[8] VP8* proteins to mucin cores 2, 4, and 6. Two doses (2 and 20 μg/ml) of the mucin cores were tested. Positive binding signals were observed for P[4] and P[8] but not for P[6]. (b) Binding of the P[II] RVs to the type 1 precursor disaccharide lacto-*N*-biose (Galβ1-3GlcNAc). Low binding signals were detected for all four P[II] RVs. (c to e) Binding of the P[II] RV VP8* proteins with the type 1 HBGAs LNT, LNFPI, and LNDFPI. The P[6] and P[19] VP8* proteins showed positive binding to the type 1 tetrasaccharide LNT (Galβ1-3GlcNAcβ1-3Galβ1-4Glc) (c) and the LNFPI pentasaccharide (Fucα1-2 Galβ1-3GlcNAcβ1-3Galβ1-4Glc) (d) with and without the terminal fucose modification (secretor), respectively. Neither of the P[4] and P[8] VP8* proteins bound the LNT and LNFPI oligosaccharides. (e) Adding a Lewis fucose to LNFPI results in the LNDFHI hexasaccharide (Fucα1-2 Galβ1-3(Fucα1-4)GlcNAcβ1-3Galβ1-4Glc), which led to negative P[6] and P[19] VP8* binding but positive P[4] and P[8] VP8* binding. The mean *A*_450_s from three replicates with standard deviations are shown.

Additional binding studies for other P[II] RVs revealed similar but genotype-specific binding properties among the three major human RVs (P[4], P[6], and P[8]) and the P[19] RVs. Like the P[19] RVs, all of the other three P[II] RVs recognized the type 1 HBGA disaccharide precursor with low affinities, while VP8* proteins of P[4] and P[8], but not of P[6], also recognized mucin cores 2, 4, and 6 (Fig. 9a and b). Since all these three major human RVs previously were shown to bind to the type 1 HBGAs (23), we performed assays comparing the binding results with those for P[19] on three type 1 HBGA glycans (LNFPI, LNT, and LNDFHI) with or without the secretor (H) and/or Lewis fucoses. The P[6] and P[19] RVs recognized LNFPI and LNT, with the former containing the H fucose, while P[4] and P[8] RVs recognized the hexasaccharide LNDFHI, which contains both H and Lewis fucoses (Fig. 9c to e). These data plus the conserved binding interface described above suggest a potential selection pressure in the maintenance of the P[II] RVs by the host mucin cores and/or type 1 HBGAs. However, observations that additional saccharide residues changed the RV binding outcomes may explain the diverse host ranges for P[II] in different human populations and animal species.

### P[10] and P[19] VP8*s shared nearly identical glycan array and saliva binding profiles.

**FIG 10:**
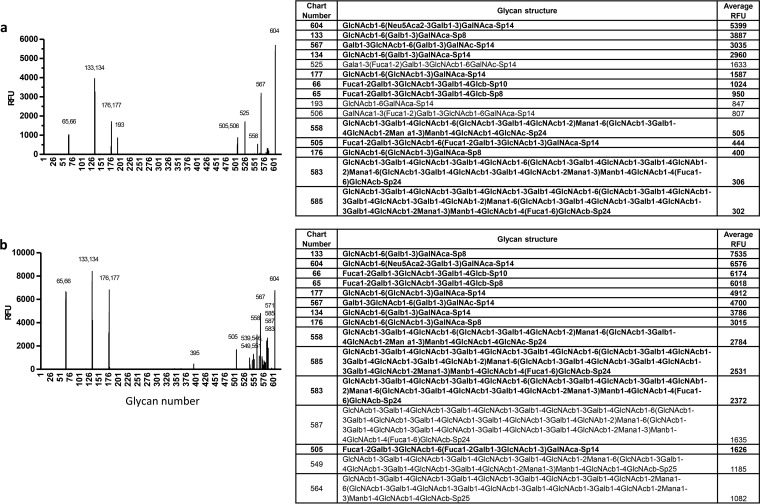
P[10] from P[I] showed a glycan binding pattern similar to that of P[19] VP8*. A glycan array analysis was performed for P[10] in comparison with P[19]. Relative fluorescent units (RFU) of GST-VP8* at 200 μg/ml binding to 610 glycans are shown, where RFU corresponds to the strength of binding to individual glycans. The identities of the 610 glycans in the array are available at http://www.functionalglycomics.org/static/consortium/resources/resourcecoreh17.shtml. The P[10] VP8* (b) revealed a binding profile similar, with a dozen identical, highly reactive glycans, to that of P[19] VP8* (a). The top 15 ranked glycans for each of the two genotypes are shown on the right. Thirteen of the 15 glycans (in bold) were shared by P[19] and P[10]. The error bars represent the standard deviations from four replicate wells.

To further study the genetic relationship between P[10] and P[19] RVs, we also performed glycan array and *in vitro* binding assays of P[10] as well as other three randomly selected P[I] RVs (P[13], P[15], and P[23]). Nearly identical saliva binding (Fig. 2c and d) and glycan array binding (Fig. 10) profiles have been observed between the P[10] and P[19] RVs, while the other tested P[I] RVs revealed completely different glycan binding profiles (data not shown). This result suggests strong selection pressures on the P[10] and P[19] genotypes by a common host factor(s), such as the mucin cores and type 1 HBGAs, which may be responsible for their common host range.

## DISCUSSION

Three major P[II] RV genotypes, P[4], P[6], and P[8], cause the vast majority (>95%) of human infections worldwide (45). Interestingly, the fourth P[II] genotype, P[19], unlike the others, is commonly found in animals (porcine) but rarely in humans (46–50). In this study, we found that P[19] VP8* binds human milk, human saliva, and porcine mucins. P[19] VP8* revealed a significantly lower profile of binding to human saliva than the highly prevalent P[4] and P[8] RVs, consistent with the low prevalence of P[19] RVs in humans observed so far. We further demonstrated that the P[19] RVs recognize the mucin cores (2, 4, and 6) and type 1 HBGA precursors as the minimal functional units. However, further modifications of these glycans with additional saccharides, such as the A, B, H, or Lewis epitopes, and even SA, could influence and further define the binding outcomes. Interestingly, this unique binding property is shared with other P[II] RVs, supported by the observation that all the other P[II] RV genotypes also recognize the mucin core and type 1 HBGA-related sequences while exhibiting a genotype-specific difference.

Specifically, all of the A, B, and H epitopes appear to be involved in P[19] RV binding, while the addition of the Lewis epitope (1,4-linked fucose) to these type 1 HBGA glycans completely blocked P[19] binding (Fig. 3). This type of epitope blocking or masking effect may explain why the P[19] RVs rarely infect humans; 90% of the general population are Lewis epitope positive, leading to Lewis epitope-negative type 1 HBGA phenotypes in only 10% of the general population (45). On the other hand, the requirement of the Lewis epitope for binding also seemed to play an important role in the high prevalence of the P[4] and P[8] RVs in humans, which was suggested by their common recognition of Lewis b (Le^b^) antigens containing the Lewis epitope (23). A strong association between Lewis epitope status and P[4]/P[8] RV infection has also been reported, where P[8] infection occurred only in Lewis epitope-positive children (31). In the current study, we further demonstrated the importance of Lewis epitopes for these two genotypes by their binding to the hexasaccharide LNDFHI (Fig. 9), which contains the Lewis epitope, but not to the type 1 glycans LNFPI and LNT, which lack the Lewis epitope.

The differences in Lewis epitope involvement for P[II] RV binding may also play a role in human versus animal host ranges, as some animal species, such as mice and pigs, lack Lewis antigens (51; our unpublished data), and the differences could explain why P[4] and P[8] RVs rarely infect animals. On the other hand, the infection of P[19] and P[6] RVs in animals may be due to their lack of Lewis epitopes, leaving the mucin cores and/or the type 1 HBGA precursors readily available to bind these genotypes. As an alternative hypothesis, it has been proposed that shared HBGAs are responsible for cross-species P[11] RV transmission between humans and animals, since P[11] recognizes evolutionarily conserved type 2 HBGA precursors or intermediate products (22).

The P[6] RV did not bind the mucin core glycans and bound poorly to the human adult saliva (23), but it recognized the pentasaccharide LNT, containing the type 1 HBGA sequences without H and Lewis epitopes, and the hexasaccharide LNFPI, without Lewis epitopes (Fig. 9). This result is consistent with those of another P[6] RV (strain RV-3) that bound a group of long glycans containing the type 1 HBGA terminal sequences (with or without the H epitope but without the Lewis epitope) and an extra Lewis x side chain (32). In addition, a negative association between P[6] RV infection in children and the presence of the Lewis gene has been reported (31). Furthermore, in the current study, we also observed an age-specific pattern of binding of P[6] RVs to saliva samples from neonates and young infants (data not shown), which is reminiscent of the age-specific host ranges in neonates and young infants found for the P[11] RVs (24, 26). Since P[6] RV infection appears to be independent of the Lewis epitope, we hypothesize that P[6] RVs may share a mechanism for age-specific host ranges similar to that of the P[11] RVs, by recognizing the HBGA precursor and/or intermediate products that may be developmentally regulated in neonates and young infants.

The finding of nearly identical saliva and glycan binding profiles between P[10] and P[19] suggests a potential evolutionary connection between P[10] and P[19] RVs, which may be selected by a common host factor such as the mucin cores and/or type 1 HBGAs. The conserved amino acids of the deduced binding interface, particularly the exclusively conserved residue W175, between P[10]/P[12] and P[19] and other P[II] RVs (P[4], P[6], and P[8]) further supports this hypothesis. Interestingly, P[10] and P[19] have very similar host ranges, both pigs and humans, while the majority of other P[I] RVs exclusively infect animals. This observation in our view raises concern regarding the strategy to develop live, attenuated animal RV reassortant vaccines for humans, because these animal RVs, such as the bovine (P[5]) and sheep (P[15]) RVs (52, 53), may not replicate in the human intestine due to a lack of proper host receptors. Future studies to clarify this concern are urgently required.

Our study raises additional issues that need to be addressed in the future. For example, based on the binding properties of P[19] and other P[II] RVs, we noted a potential binding specificity switch from simple mucin core glycans to more complex type 1 HBGAs, leading to host range changes between animal (P[I]) and human (P[II]) RVs and among P[II] RVs in different human populations. However, direct evidence, including supporting structural data, for such host range switching and variations remains lacking. While mucin core 2 is commonly found in a variety of cells and tissues, mucin core 4 and 6 sequences were found only in a very limited type of mucin, that is, in human intestinal mucins (54). Thus, future studies to determine the role of mucin cores versus type 1 HBGAs in human RV host ranges are necessary. Finally, during our characterization of P[19] and other P[II] and P[I] RVs, we noted that the RV-glycan interactions are far more complicated than previously thought. While the minimal binding units of the HBGA and/or mucin core sequences are essential for binding, further terminal or internal side chain modifications, the lengths of the glycan backbones, and repeated sequences could strongly influence or change the binding affinities and/or specificities and, therefore, the final binding outcomes. Thus, future studies are needed to define the precise amino acids within the binding interface that are responsible for specific interactions between each sugar residue and the individual P genotypes characterized in this study.
